# The Use of Winter Water Temperature and Food Composition by the Copepod *Cyclops vicinus* (Uljanin, 1875) to Provide a Temporal Refuge from Fish Predation

**DOI:** 10.3390/biology10050393

**Published:** 2021-05-01

**Authors:** Jong-Yun Choi, Seong-Ki Kim

**Affiliations:** National Institute of Ecology, Seo-Cheon Gun 325-813, Chungcheongnam Province, Korea; skkim@nie.re.kr

**Keywords:** fish predation, prey defense strategy, predator–prey interactions, copepods, food algae, biodiversity

## Abstract

**Simple Summary:**

Predator avoidance mechanisms play a critical role in the survival and stable population growth of prey. Here, we describe a new defense strategy for *Cyclops vicinus*, which is vulnerable to fish predation. Long-term data (January 2014 to February 2019) showed that *C. vicinus* was abundant in winter when the foraging activity of fish was lower. This pattern was reversed in spring, summer, and autumn. *C. vicinus* is consumed frequently by fish because it has a body size larger than that of other cyclopoid copepods (*Mescyclops leuckarti* and *Thermocyclop* sp.). In this respect, winter formed a seasonal refuge when *C. vicinus* populations could grow efficiently. In addition, there was an abundant phytoplankton presence (*Cyclotella* sp. and *Rhodomonas* sp.) in winter. These species formed a food source that supported the population growth of *C. vicinus*. The evolution of the predator avoidance mechanisms of prey contributes significantly to the security of local biodiversity and the stability of the freshwater food web.

**Abstract:**

Frequent predation induces various defense strategies in prey, including morphological changes or migration patterns in zooplankton. We hypothesized that the winter dominance of *Cyclops vicinus* in the Upo Wetlands, South Korea, is an evolved temporal defense mechanism to avoid fish predation. Long-term data (2014–2019) showed that fish consumed the most cyclopoid copepods from spring to autumn. *Lepomis macrochirus* preferentially consumed *C. vicinus*; thus, *C. vicinus* density was lower from spring to autumn. However, *C. vicinus* was abundant in winter when fish consumed fewer copepods. Nauplii density began to increase in late autumn (October–November), and their population growth was fueled through consumption of *Cyclotella* sp. and *Rhodomonas* sp. Culture experiments showed that *Cyclotella* sp. contributed more to the growth stage (copepodite or subadult) after nauplii than *Rhodomonas* sp. *C. vicinus* density was lower in the winters of 2013 and 2016 when the densities of these phytoplankton prey species were lower. In summary, although winter conditions were suitable for copepod survival and population growth, *C. vicinus* relied heavily on the diversity and species composition of its food sources. The winter dominance of *C. vicinus* could increase regional biodiversity and contribute significantly to the stability of the freshwater food web.

## 1. Introduction

Predation is an important factor that affects local population size, species diversity, and biological interactions [1,2]. Imbalances in ecological food webs are mainly caused by changes in biological interactions (i.e., predation) that increase or decrease local population density at other trophic levels. These population variations can be influenced by internal environmental factors, such as physicochemical parameters [3,4], as well as external factors such as the introduction of exotic species or human disturbance (e.g., fishing and recreation activity [5]). Various empirical studies have focused on analyzing population sizes or monitoring distribution patterns in relation to ecosystem function or structure [6,7]. Predators’ feeding activities are determined by their food searching ability and the frequency of encountering their prey [8,9]. Prey populations that are concentrated in relatively restricted habitats are more likely to have frequent contact with predators [10]. In this regard, freshwater animals have unfavorable environmental conditions, compared to terrestrial or oceanic animals. Their spatial range is restricted by the intermittent supply of water in freshwater ecosystems such as streams, wetlands, and ponds, and they also occur in fragmented habitats [11]. Therefore, freshwater ecosystems have more frequent and excessive prey‒predator interactions than other ecosystem types (e.g., terrestrial ecosystems) [12]. Hence, biological interactions such as predation play critical roles in maintaining the function and balance of freshwater ecosystems.

In freshwater ecosystems, foraging activity by predator has led to the development of different defense strategies by prey species. In terms of the evolutionary arms race [13,14], the evolution of a prey species is matched by the development of more efficient predation; however, the defense strategies of freshwater prey are sometimes more effective than the adaptation of predators. Among the various freshwater flora, zooplankton communities have efficient defense strategies for predator avoidance. The consumption of zooplankton as a main food source for invertebrates (e.g., dragonfly larvae, mosquito larvae, and fly larvae) and fish is sufficient to stimulate the development of defense strategies to avoid predators [15,16]. For example, in shallow wetlands, zooplankton communities are mainly abundant in areas with a high aquatic macrophyte cover, because prey location by fish is hindered by their leaves and stems [17,18,19]. In particular, wetlands can support higher densities and species diversity of cladoceran communities than other freshwater ecosystems (e.g., rivers or reservoirs) due to the active use of aquatic macrophytes by cladocerans that are vulnerable to fish predation [20]. Similarly, the littoral swarming of *Moina micrura* is a behavioral mechanism for avoiding predation [21]. Littoral areas are effective refuges for zooplankton because they are shallow, and the foraging activities of predators are restricted by disturbances on land. Furthermore, the lack of light and the low dissolved oxygen in the hypolimnion of lakes and reservoirs restrict fish distribution; thus, the deeper layer can be used as shelter by some zooplankton species (*Daphnia* spp.; [22,23,24]). Empirical studies explain that the use of refuges by zooplankton is more of an evolutionary mechanism than one with morphological changes, such as increases in length of the head or spin [25,26].

The refuges for zooplankton introduced in previous studies are mainly created by spatial differences in their environmental characteristics. These spaces provide an advantage for zooplankton because chemical factors (such as low dissolved oxygen) or physical factors (such as shallow depths or complex construction of aquatic macrophyte leaves or stems) strongly restrict predator distributions [27,28]. However, a concentrated predator distribution in refuge spaces can lead to evolution in predators’ foraging abilities or an increased density of predators that can efficiently capture food sources in the area. For example, bluegill sunfish (*Lepomis macrochirus*), an exotic species, established itself in the wetlands of South Korea by occupying mainly vegetated areas where other predatory fish are restricted [29]. *L. macrochirus* is capable of efficient foraging activity, even with a moderate aquatic macrophyte abundance [30]. Largemouth bass (*Micropterus salmoides*) was introduced around the same period as *L. macrochirus* and interfered with their establishment by consuming *L. macrochirus*; however, the high quantity of aquatic macrophytes that is supported by Korean wetlands greatly hindered the predatory activities of *M. salmoides* and contributed to the stable establishment of *L. macrochirus* [29]. Although various empirical studies have suggested that vegetated areas are highly effective as refuges for zooplankton because plants negatively affect the fish foraging activities [31,32,33], vegetated areas with larger *L. macrochirus* populations only have a minor refuge effect [29]. The presence of predators in existing refuges requires novel defense strategies that are clearly different from the existing strategies of prey such as zooplankton. These can include previously mentioned approaches such as migration behavior or habitat preferences or may take a completely new form. Copepods and cladocerans are frequently consumed by fish, and therefore, their populations tend to be sensitive to predation [34].

In this study, we report on a new defense strategy by which copepod communities avoided predators. Copepods have the position as primary consumers in freshwater food webs, and since they are a food source for fish and invertebrates, various defense strategies are needed to avoid predators. Previous studies suggest that behavioral reactions or distribution changes such as jumps or accelerated movement [35], migration patterns [36,37], bottom-layer distributions [38], and aquatic macrophyte utilization [39] are predator avoidance responses of copepods. We hypothesized that the winter distribution of copepods observed in the Upo Wetlands is more of an evolutionary strategy, stemming from their predator avoidance mechanisms. Since its introduction in the 1970s, high copepod consumption by *L. macrochirus* has been reported in various studies [40,41], which is assumed to have induced new predator avoidance strategies. However, information on the winter distribution of copepods or their associated temporal refuge use in previous limnological studies is insufficient. We suggest that understanding the predator avoidance mechanisms of copepods is important for securing local biodiversity and will greatly contribute to the stabilization of freshwater food webs.

The aims of this study were to elucidate (1) the seasonal changes in cyclopoid copepod communities in relation to environmental variations, (2) the consumption pattern of copepods by fish predators, and (3) the influence of winter growth on copepods in relation to their food sources (i.e., phytoplankton). To address these objectives, we surveyed the environmental variables, cyclopoid copepods, fish, and phytoplankton in the Upo Wetlands in southeastern South Korea. During this long-term study (January 2013‒February 2019), we analyzed the seasonal responses of copepods to fish predation and food sources (i.e., phytoplankton). We found high copepod consumption by fish during all seasons except winter, in addition to a close relationship between the winter distribution of cyclopoid copepods and the seasonality of some phytoplankton species (*Cyclotella* sp. and *Rhodomonas* sp.).

## 2. Materials and Methods

### 2.1. Study Description

**Figure 1:**
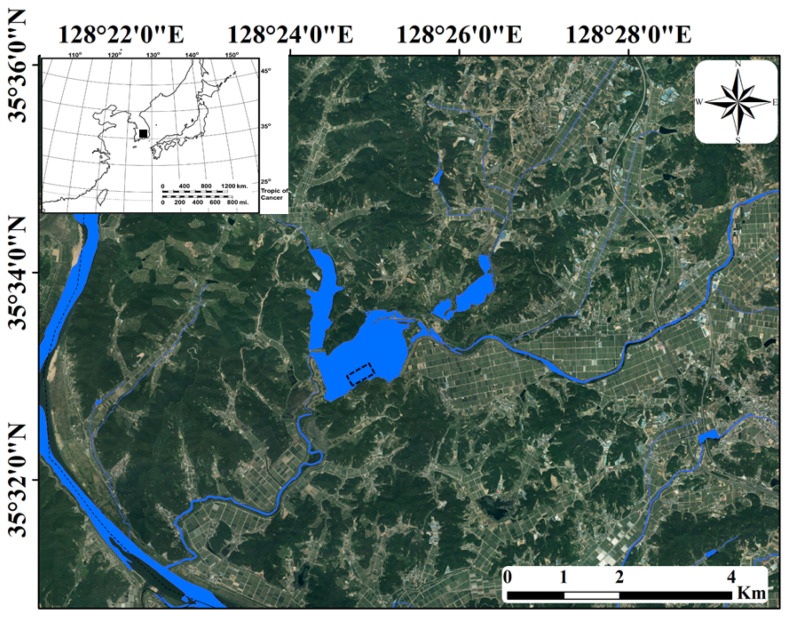
Upo Wetlands in southeastern South Korea. The sampling point is shown by the dotted rectangle. The inset map indicates the Korean Peninsula, and the study site is indicated by the black square.

The Upo Wetlands are riverine wetlands located in the middle and lower reaches of the Nakdong River (Figure 1). The majority of this area has a poor drainage capacity and is covered with a large number of wetlands owing to flooding by the Nakdong River in southeastern South Korea or major tributary streams. Summer-concentrated rainfall leads to frequent flooding in the region and negatively affects the distribution and population growth of various biological communities [42]. However, in other seasons (spring, autumn, and winter), when less rainfall occurs, a relatively stable environment is maintained. Topyeong Stream is the main water source of the Upo Wetlands, and it passes through the wetlands to flow into the middle and lower reaches of the Nakdong River. The Upo Wetlands are divided into four large and small wetlands (Upo, Mokpo, Sajipo, and Sojibeol), of which Upo (1.28 km^2^) is larger than the combined area of the other three wetlands (1.05 km^2^). In the past, the Upo Wetlands were intermittent wetlands with very large water level changes depending on flooding in Topyeong Stream; however, it has maintained its current form and water depth since the construction of an embankment [43]. The water depth of the Upo Wetlands ranges from 0.2 m in the littoral zone to 1.2 m in the center. The shallow and nutrient-rich waters provide suitable conditions for aquatic macrophyte growth. The main aquatic macrophytes observed in the Upo Wetlands are *Phragmites australis*, *Paspalum distichum*, *Zizania latifolia*, *Spirodela polyrhiza*, *Salvinia natans*, *Trapa japonica*, *Ceratophyllum demersum*, and *Hydrilla verticillata*, which cover the water surface from spring to autumn. The four distinct seasons in the middle and lower reaches of the Nakdong River lead to the seasonal growth and dynamic succession of various biological communities, including aquatic macrophytes. Water temperatures range from 10 to 28 °C from spring to autumn, providing suitable conditions for the growth of various aquatic organisms; however, in winter, a low water temperature of 1.6 °C hinders the growth of most aquatic organisms.

### 2.2. Monitoring Strategy

Biweekly monitoring was conducted for 7 years (January 2013‒February 2019). Prior to the investigation, we selected six sampling points (1 m × 1 m quadrats) within the wetland characterized by similar environmental variations (i.e., plant species composition, water depth, and physicochemical factors). Three quadrats were used to investigate environmental variables and cyclopoid copepods. The remaining three quadrats were used to collect biological items for stable isotope analysis, which was performed to identify the potential food source for *C. vicinus*.

In order to explain the seasonal and yearly distribution of cyclopoid copepods in relation to environmental variables in the Upo Wetlands, we used rainfall data during field survey time (January 2013‒February 2019) obtained from the Korea Meteorological Administration (KMA, Seoul, South Korea; http://www.kma.go.kr access on 8 January 2021), which was collected from Hapcheon Station (i.e., the closest gauging station to the study site).

Environmental variables (water depth, water temperature, pH, dissolved oxygen concentration [DO], conductivity, and turbidity) were measured at the three sampling points. Water depth was measured using a 1.5 m steel ruler. Water temperature and DO were recorded using a DO meter (Model 58; YSI Inc., Yellow Springs, OH, USA). A conductivity meter (Model 152; Fisher Scientific, Hampton, NH, USA) and an Orion 250A pH meter (Orion Research Inc., Boston, MA, USA) were used to determine the conductivity and pH, respectively. Turbidity was measured using a turbidimeter (Model DRT 100 B, HF Scientific, Inc., Fort Meyers, FL, USA). For cyclopoid copepod enumeration, we collected 10 L water samples using a 10 L column water sampler (length: 20 cm; width: 30 cm; height: 70 cm) from each quadrat. The sampler was placed vertically into the water to collect copepods from the entire water column of the quadrat. Water samples were filtered through a 70 µm mesh plankton net and the filtrate was preserved in formalin (final concentration: 4% formaldehyde [44]). Copepod enumeration and identification at the genus or species level were performed using a microscope (ZEISS, Model Axioskop 40; 200× magnification), with identification based on the classification key by Mizuno and Takahachi [45].

In order to understand the influence of the winter distribution of copepods on fish predation and food sources, we investigated the seasonality of fish and phytoplankton. Fish were collected using a cast net (7 mm × 7 mm) and scoop net (5 mm × 5 mm) along 200 m transects in each season (winter [January], spring [May], summer [August], and autumn [November]) from January 2014 to February 2019. Fish and copepods were collected on different days. The cast net and scoop net were used for 30 min and 20 min, respectively. Fish samples were identified to the species level according to Kim and Park [46] and the classification system of Nelson et al. [47]. Fish species that were difficult to identify in the field were fixed using a methanol–formaldehyde solution (3:1) and were subsequently identified in the laboratory. Furthermore, to identify the food consumption tendency of fish, we immediately fixed the gut of the dominant fish species (*L. macrochirus*) collected in each season using a methanol–formaldehyde solution (3:1). We identified and counted all food items (copepods, branchiopods, isopods, dipterans, odonatans, and young fish) in the gut contents of *L. macrochirus*, and the abundance of each food item was calculated as the abundance per weight of the gut. We used 30 *L. macrochirus* individuals in each season to identify food consumption patterns.

For phytoplankton enumeration, we collected 1 L surface water samples from January 2013 to February 2019 and fixed the samples with neutral Lugol’s solution. All phytoplankton samples were kept in a laboratory refrigerator at 4 °C and analyzed as soon as possible after collection. Phytoplankton identification and enumeration were carried out using a microscope (ZEISS, Model Axioskop 40), and 1000× magnification was used for species identification. After allowing 48 h for phytoplankton samples to deposit, the samples were concentrated to 30 mL using a siphonage method and stored at 4 °C. Then, each sample was added to a 0.1 mL counting chamber (20 × 20 mm) and phytoplankton species were identified and counted according to Hu et al. [48]. The examined phytoplankton were Bacillariophyceae, Chlorophyceae, Cryptophyceae, Cyanophyceae, Euglenophyceae, and Dinophyceae.

### 2.3. Stable Isotope Analysis

Stable isotope analysis was implemented to identify the potential food items of *C. vicinus* groups (nauplii, copepodites, and adults). Suspended particulate organic matter (SPOM, i.e., free or uncomplexed organic matter > 50 µm; predominantly phytoplankton), epiphytic particulate organic matter (EPOM, organic matter > 50 µm attached to the stem and leaves of aquatic macrophytes; predominantly periphytic diatoms), benthic particulate organic matter (BPOM, organic matter > 50 µm collected on the bottom surface; predominantly periphytic diatoms), and *C. vicinus* groups (nauplii, copepodite, and adults) were sampled in each winter month (December, January, and February) from 2014 to 2019. These samples were collected three times per month in addition to the regular monitoring program. We collected 5 L of surface water per sample (*n* = 4). To process the SPOM samples, micro- or macroinvertebrates were initially removed using a 32 µm mesh size plankton net, and the water samples were then filtered through GF/F glass fibers (0.45 µm; pre-combusted at 500 °C for 2 h). The surfaces of the submerged parts (stems, leaves, and roots) of the macrophytes present at the sampling points were gently brushed in a tank filled with distilled water to retain the EPOM. The BPOM was obtained by carefully floating the bottom substrate. Care was taken not to mix organic matter other than the bottom surface in this process. Similar to the SPOM processing step, plant debris and zooplankton were removed using a 32 µm mesh size plankton net. To obtain *C. vicinus* groups, a total of 10 L water was filtered through a plankton net (70 µm mesh size) using a 10 L column sampler (length: 20 cm; width: 30 cm; height: 70 cm). To prevent injury in dense samples of *C. vicinus* groups, filtration using a plankton net was conducted before the detailed separation of the *C. vicinus* group in the laboratory. Copepod groups were sorted into nauplii, copepodites, and adults using fine tweezers and spoids. In January and February, when the nauplii and copepodites densities were low, only adults were used for stable isotope analysis.

The POM samples (SPOM, EPOM, and BPOM) were treated with 1 mol L^−1^ HCl to remove inorganic carbon. The samples were then rinsed with deionized distilled water to remove the acid. All samples were freeze dried and then ground with a mortar and pestle. Powdered samples were frozen at −70 °C until analysis. Carbon and nitrogen isotope ratios were determined using continuous-flow isotope mass spectrometry (CF-IRMS, Model-ISOPRIME 100; Micromass Isoprime, GV Instruments Ltd., Manchester, UK). Prior to analysis, the samples were placed overnight in a sealed CF-IRMS through which 99.999% He was flowing at a few mL/min. Instrument linearity (dependence of δ^13^C and δ^15^N on signal amplitude at the collectors) was tested daily and confirmed to be <0.03‰/nA over the 1–10 nA range. 100 (±10) μg silver-encapsulated cellulose samples (i.e., no carbon added to samples inside capsules), producing approximately 4–6 nA signal at the collectors, were loaded in a 99-position zero-blank CF-IRMS and converted to a mixture of carbon monoxide, carbon dioxide, water, and hydrogen gases over glassy carbon chips in a quartz tube at 1080 °C, within a stream of 99.999% carrier He flowing at 110 mL/min. Data are expressed as the relative (‰) difference per mL between the sample and the conventional standards of Pee Dee Belemnite (PDB) carbonate for carbon and atmospheric N_2_ for nitrogen, according to the following equation:

δ X (‰) = [(R_sample_/R_standard_) − 1] × 1000,

where X is ^13^C or ^15^N, and R is the ^13^C:^12^C or ^15^N:^14^N ratio. A secondary standard of known relationships to the international standard was used as the reference material. The standard deviations of δ^13^C and δ^15^N for 20 replicate analyses of the peptone (δ^13^C = −15.8‰ and δ^15^N = 7.0‰, Merck) standard were ±0.1 and ±0.2‰, respectively.

### 2.4. C. vicinus Growth Experiments

For the *C. vicinus* growth experiments, *C. vicinus* and two food algae species (*Rhodomonas* sp. and *Cyclotella* sp.) were collected from the sampling point in the Upo Wetlands, where field investigations were conducted. We selected the collection period considering the seasonal variability of each target species in the field. *C. vicinus* was obtained using 150 µm mesh plankton nets during late autumn (late October‒early November 2016). In the laboratory, adult females (length: 1.2–1.8 mm) with eggs were sorted and anesthetized with carbonated water. Eggs were removed with a dissecting pin and maintained in small dishes containing 25 mL of aged tap water at room temperature (18–20 °C). Nauplii that hatched after approximately 2 days were transferred within 12 h of hatching into 500 mL sterilized beakers filled with 500 mL distilled water.

*Rhodomonas* sp. and *Cyclotella* sp. were separated from phytoplankton samples collected in November 2016 and January 2017, respectively. To increase the abundance of the target species, the water samples collected from the Upo Wetlands were filtered using a 32 µm mesh plankton net. *Rhodomonas* sp. and *Cyclotella* sp. were extracted from phytoplankton samples using a microscope (ZEISS, Model Axioskop 40, Jena, Germany). To provide stable food for *C. vicinus* growth, *Rhodomonas* sp. and *Cyclotella* sp. were maintained using a plant growth chamber (Eyela FLI-301N, Tokyo, Japan), with 50 photon flux density (µmol·m^−2^·sec^−1^), and a 12 L:12 D light: dark cycle in Bold’s Basal Medium [49]. *Rhodomonas* sp. and *Cyclotella* sp. were cultured at 10 °C and 5 °C, respectively, considering their seasonal distribution patterns within the Upo Wetlands.

Three different food concentration conditions for each algal species (1 × 10^4^ cells mL^−1^, 1 × 10^5^ cells mL^−1^, and 4.5 × 10^5^ cells mL^−1^) were established according to Hopp and Maier [50] to identify the influence of the quantity of different food sources on *C. vicinus* growth. A concentration of 4.5 × 10^5^ cells mL^−1^ was considered sufficient for *C. vicinus* survival and population growth (22.5 mg CL^−1^), and 1 × 10^4^ cells mL^−1^ was insufficient (0.5 CL^−1^). The required quantity of algae was calculated from the relationship between algal density and carbon content [51]. We used 250 mL beakers for the entire experiment, and the amount of algal food was adjusted to each food condition. The culture medium used in the *C. vicinus* growth experiment was prepared such that water samples obtained from the sampling points where *C. vicinus* was collected were filtered twice using 0.45 µm mesh filter paper. We determined that there were no algae or organic matter available as food for *C. vicinus* in the filtered water using a microscope (ZEISS, Model Axioskop 40). To avoid pH changes owing to photosynthesis, we stored filtered water in the refrigerator (2 °C) and removed it just prior to the experiment. Growth experiments were conducted using a plant growth chamber (Eyela FLI-301N, Japan), with a 50-photon flux density (µmol·m^−2^·sec^−1^), and a 12 L:12 D light–dark cycle. We placed 20 beakers containing each food concentration, with 50 individual nauplii placed in each 500 mL beaker. Each day during the experiment, we transferred the nauplii and copepodites to a fresh culture medium (filtered field water) that met each food condition, before providing food algae, to maintain the supply of algae under each condition. Growth experiments were conducted for approximately 80 days, and the surviving individuals in five randomly selected beakers were counted every 5 days. We calculated the survival rate (%) as the number of surviving individuals on each day/total number of individual nauplii (i.e., 50) in each beaker.

### 2.5. Data Analysis

We used nonmetric multidimensional scaling (NMDS) to examine the distribution patterns of the cyclopoid copepod groups due to environmental variations. The NMDS ordination plots were generated based on the Euclidean distance, and the goodness of fit was assessed in terms of the loss of stress. Each variation was log transformed after being assessed for normality using the Shapiro–Wilk test. Rare copepod species with observed densities of <50 ind.L^−1^ per year were excluded from the ordination analysis, leaving five cyclopoid copepod groups (*C. vicinus*, *Mesocyclops leuckarti*, *Thermocyclops* sp., copepodite, and nauplii) for final analysis. The stress value for the two-dimensional solution was 0.156, which was lower than the generally accepted maximum stress value of <0.2 [52]. The significance of the fitted vectors was assessed using 3000 permutations, with *p* < 0.05 considered significant. NMDS ordination was conducted using the R package “vegan” (version 2.5–3 [53]).

For statistical analyses of the growth experiment, we applied a one-way nested ANOVA (two-tailed, a = 0.05) to explain the survival and growth patterns under different food conditions. Although we prepared 20 replicates (beakers) for each experimental group (three food conditions), pseudoreplication required careful consideration (i.e., data homogeneity between replicates for each experimental group needed to be ensured) [54]. Therefore, we set the different food concentration conditions as the primary factors and the 20 beakers as nested subgroups for each treatment.

## 3. Results

### 3.1. Environmental Variables and Cyclopoid Copepod Distributions

**Figure 2:**
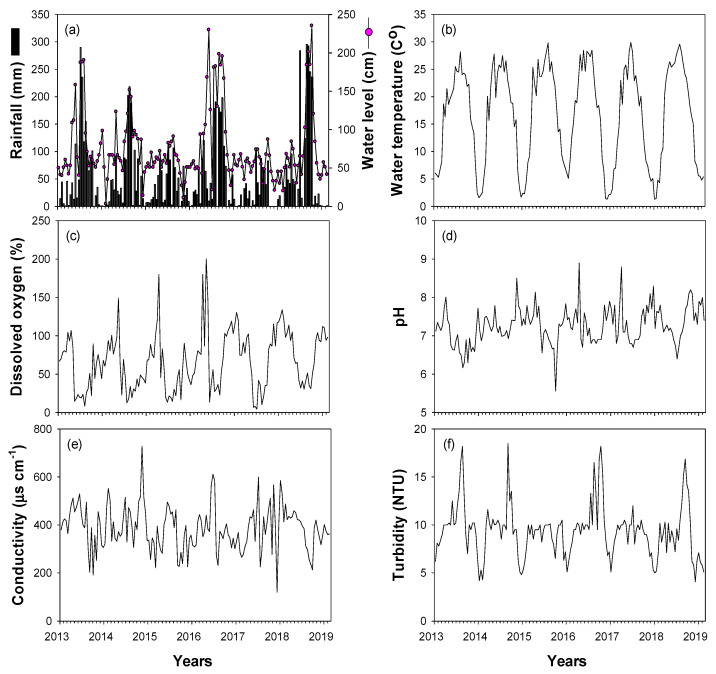
Time-series fluctuations in environmental variables for the study sites in the Upo Wetlands from January 2014 to February 2019. (**a**) Rainfall and water level, (**b**) water temperature, (**c**) dissolved oxygen, (**d**) pH, (**e**) conductivity, and (**f**) turbidity.

Most of the environmental variables (rainfall, water level, water temperature, dissolved oxygen, pH, conductivity, and turbidity) showed clear seasonal fluctuations (Figure 2). Rainfall was concentrated in summer (approximately 57% of annual mean rainfall in July–September), with an annual mean of 794 mm. High rainfall was recorded in the summers of 2013 (961 mm), 2014 (783 mm), 2016 (932 mm), and 2018 (1256 mm), whereas less rainfall occurred in 2015 (402 mm) and 2017 (430 mm). Water level and turbidity were strongly related to interannual changes in rainfall. Spring and winter water levels were relatively low, whereas water levels in summer and autumn depended on changes in summer rainfall. Turbidity was <10 NTU from spring to autumn and showed high values in summer and autumn of 2013, 2014, 2016, and 2018, when there was high summer rainfall. Water temperatures were high in summer and low in winter (November–February), while DO, pH, and conductivity showed contrasting patterns (low in summer and high in winter).

**Figure 3:**
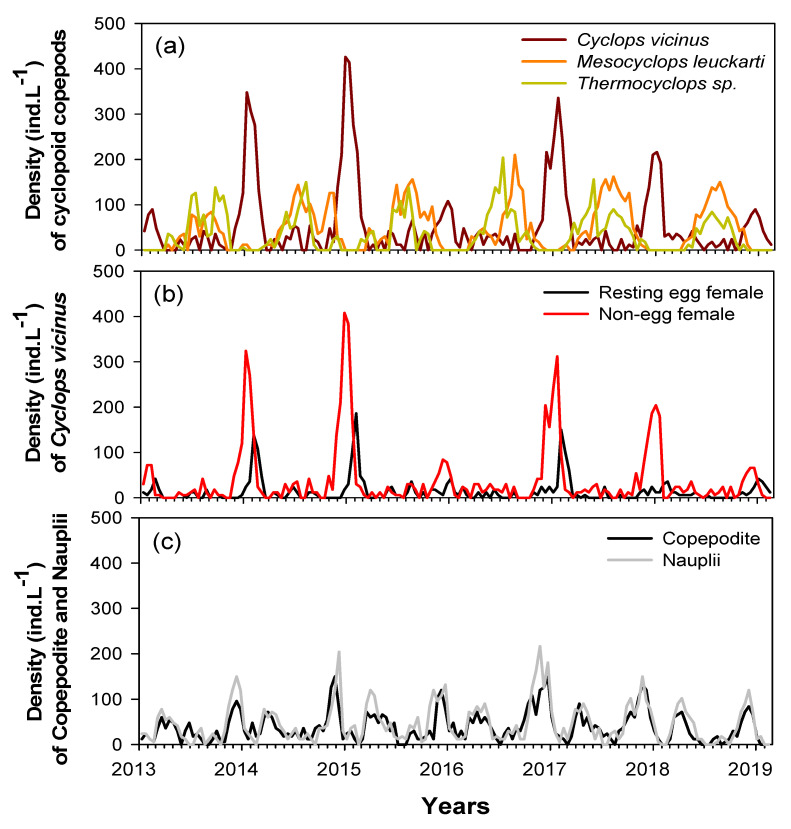
Interannual changes in abundance of cyclopoid copepods during the study period (January 2014 to February 2019) in the Upo Wetlands. (**a**) Densities of three cyclopoid copepod species, (**b**) densities of resting egg and non-egg-bearing *Cyclops vicinus* females, and (**c**) densities of copepodite and nauplii.

Five cyclopoid copepod species were identified in the study sites during the study period. *Cyclops vicinus*, *Mesocyclops leuckarti*, and *Thermocyclops* sp. were the most dominant (96%), followed by *M. fuscus* and *Eucyclops* sp. (4%). Long-term monitoring data (January 2013‒February 2019) showed different seasonal variability of the three dominant species (Figure 3). *Mesocyclops leuckarti* and *Thermocyclops* sp. were abundant in summer‒autumn (June‒October), while a high *C. vicinus* density occurred in winter (January‒February). While *M. leuckarti* and *Thermocyclops* sp. growth were sustained in summer and autumn in each year, the winter peak of *C. vicinus* differed each year. *C. vicinus* was abundant in the winters of 2014, 2015, 2017, and 2018 (mean 220 ind./L); however, densities were relatively low in the winters of 2013, 2016, and 2019 (mean 52 ind./L). Adult *C. vicinus* were dominated by a high density of females with eggs in late winter (February) and were mainly dominated by females without eggs in early and midwinter (Figure 3b). Nauplii and copepodite stages were abundant from November to December, i.e., before the winter peak of the *C. vicinus* density, and in the spring before the summer peaks of *M. leuckarti* and *Thermocyclops* sp. density (Figure 3c). Although we could not identify these nauplii and copepodite stages to a species level, based on the clear seasonal variability of the three dominant species, the nauplii and copepodites from November to December were assumed to be young *C. vicinus* life stages, while those in spring were *M. leuckarti* and *Thermocyclops* sp.

**Figure 4:**
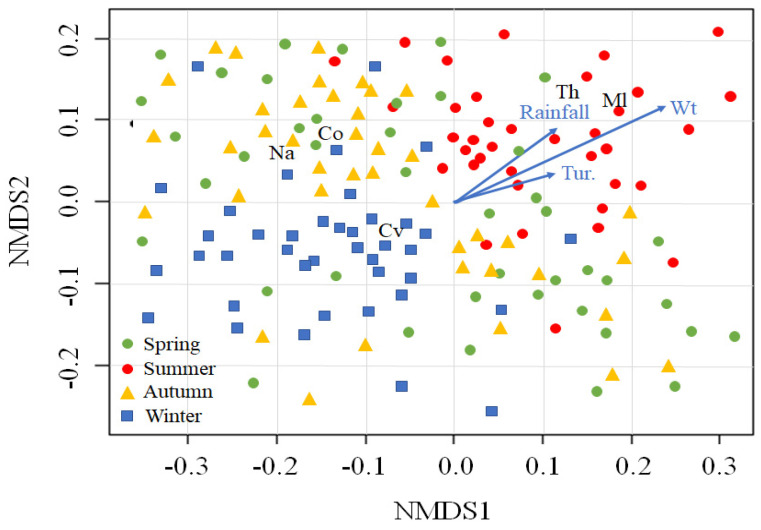
Nonmetric multidimensional scaling (NMDS) of five cyclopoid copepods groups (*Cyclops vicinus*, Cv; *Mesocyclops leuckarti*, Ml; *Thermocyclops* sp. Th; nauplii, Na; and copepodite, Co) and 160 sampling times (dots). The blue arrows indicate the associations with environmental variables. The sampling times are divided into four seasons (spring, summer, autumn, and winter). Wt, water temperature; Tur, turbidity.

We fitted the five cyclopoid copepod groups to NMDS ordination sites and selected four environmental variables that were significantly correlated with those locations (*p* < 0.05; Figure 4). *Mesocyclops leuckarti* (Ml) and *Thermocyclops* sp. (Th) were abundant in summer and were associated with higher water temperature, rainfall, and turbidity. In contrast, a high *C. vicinus* (Cv) density was found in winter, associated with lower water temperature, rainfall, and turbidity. Nauplii and copepodites distributions were mainly related to autumn.

### 3.2. Fish Predation

**Figure 5:**
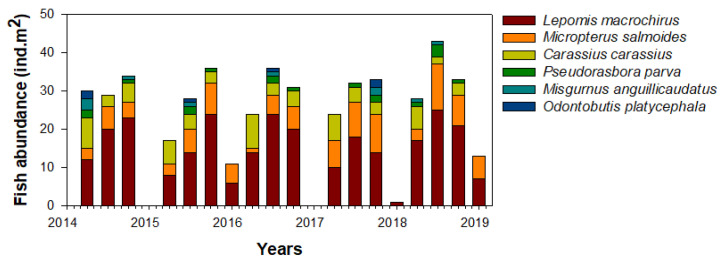
Seasonal abundance of the fish community in the study site in the Upo Wetlands (2014–2019).

We collected a total of six fish species (*L. macrochirus*, *M. salmoides*, *Carassius carassius*, *Pseudorasbora parva*, *Misgurnus anguillicaudatus*, and *Odontobutis platycephala*) from the Upo Wetlands during the study period (Figure 5). Lepomis macrochirus was the most dominant in the study site (57.7%), followed by M. salmoides (20.2%), and *C. carassius* (14.9%). The relative richness of the remaining fish species (*P. parva*, *Misgurnus anguillicaudatus*, and *O. platycephala*) was approximately 7%, although this varied over time. The fish community was abundant in summer and autumn and fluctuated every year in spring. In contrast, few fish species were present during most winter periods (i.e., except for *L. macrochirus* and *M. salmoides* collected in 2016 and 2018). The seasonal patterns of these fish communities were similar every year.

Corresponding to their seasonal distribution, the feeding activity of *L. macrochirus* mainly occurred from spring to autumn (Table 1). In winter, the collection frequency of *L. macrochirus* was low, and their food consumption was also low. The food items consumed by *L. macrochirus* were the highest in the copepod community (36%), followed by branchiopods (27%). Predation by *L. macrochirus* on the remaining four food communities (isopods, dipterans, odatans, and young fish) was relatively low. The consumption patterns of copepods and branchiopods for *L. macrochirus* showed distinct seasonal variability. In summer and autumn, copepod consumption by *L. macrochirus* was approximately twice as high as that of the branchiopods, whereas, in spring, branchiopod consumption was higher. *L. macrochirus* consumed more *C. vicinus* (49.5%) than *M. leucarti* (31.5%) and *Thermocyclops* sp. (18.9%) (Table 2).

### 3.3. Winter Food Utilization by C. vicinus

**Figure 6:**
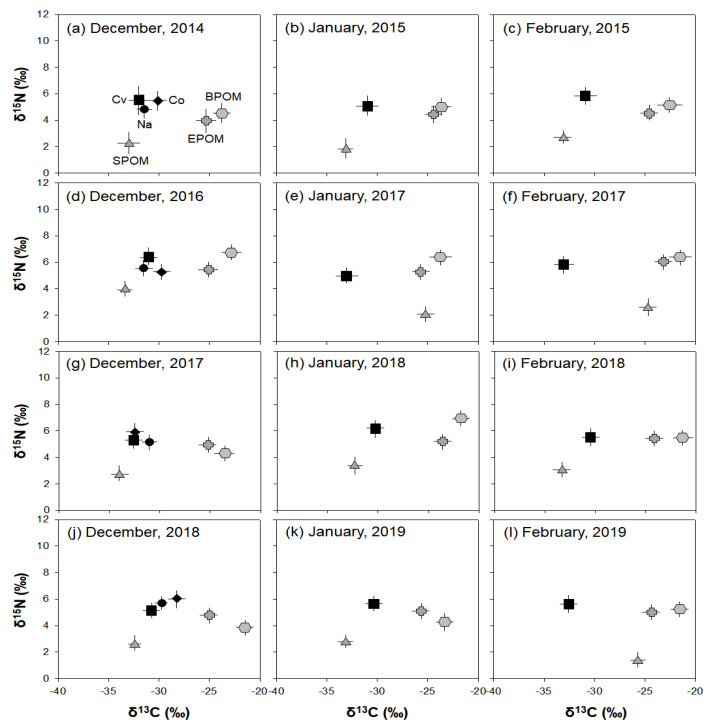
Carbon and nitrogen isotope plots of samples (*n* = 3) in winter (December–February) in the Upo Wetlands. SPOM, suspended particulate organic matter; EPOM, epiphytic particulate organic matter; BPOM, benthic particulate organic matter; Cv, *Cyclops vicinus*; Co, copepodite; Na, nauplii. (**a**) December, 2014, (**b**) January, 2015, (**c**) February, 2015, (**d**) December, 2016, (**e**) January, 2017, (**f**) February, 2017, (**g**) December, 2017, (**h**) January, 2018, (**i**) February, 2018, (**j**) December, 2018, (**k**) January, 2019, (**l**) February, 2019.

The stable isotope analysis results showed the annual consumption patterns of three potential food sources (SPOM, EPOM, and BPOM) of *C. vicinus* in winter (Figure 6). *C. vicinus* was more closely associated with SPOM than with EPOM or BPOM during most winter periods. The δ^13^C and δ^15^N values of *C. vicinus* adults reflected winter monthly changes in the δ^13^C and δ^15^N values of the food source (SPOM) and indicated a shift in the food source composition. However, the close relationship between *C. vicinus* and SPOM was rare in January and February 2017 and February 2019. During these periods, the δ^13^C value of SPOM was heavier than that of other winter periods, whereas the δ^13^C value of *C. vicinus* was similar to that of other winter periods. Nauplii and copepodites, which declined in December, also relied on SPOM. The δ^13^C and δ^15^N values of EPOM and BPOM were always higher than those of SPOM, and their relevance to *C. vicinus* was very low. The consistent contribution of SPOM to *C. vicinus* during winter indicated that *C. vicinus* mainly consumed phytoplankton.

**Figure 7:**
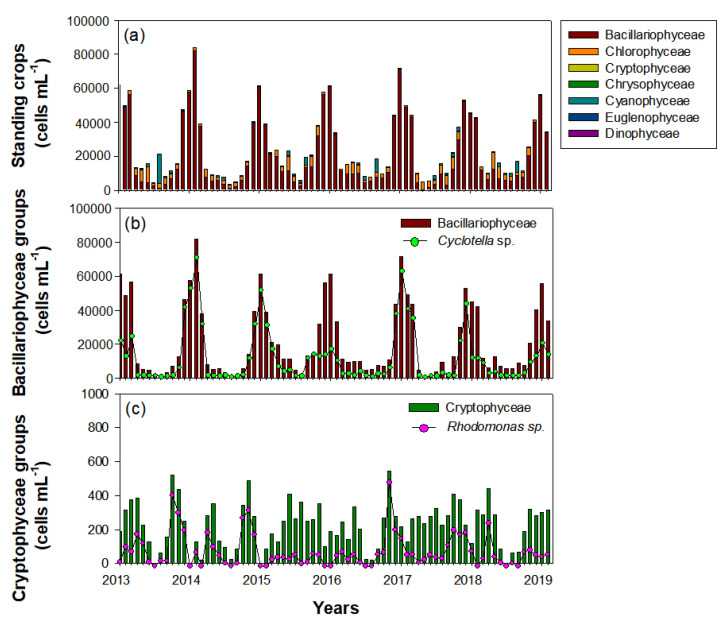
Interannual changes in standing crops of phytoplankton during the study period (January 2014‒February 2019) in the Upo Wetlands. (**a**) Total phytoplankton (including five main classes), (**b**) Bacillariophyceae groups, and (**c**) Cryptophyceae groups.

Phytoplankton in the Upo Wetlands were dominated by Bacillariophyceae (84.5%), followed by Chlorophyceae (11%) and Cyanophyceae (3.6%) (Figure 7). Thus, the total phytoplankton distribution depended on the seasonal variability of Bacillariophyceae. During the survey period, the Bacillariophyceae population began to increase in October and remained high until February, and was mainly composed of *Cyclotella* sp. During late autumn and winter (October‒February), *Cyclotella* sp. abundance was approximately four times greater than that in other seasons (spring to mid-autumn). However, during winter, *Cyclotella* sp. showed a different distribution pattern every year. The *Cyclotella* sp. abundance in the winters of 2014, 2015, and 2017 (i.e., January‒February) accounted for the majority of the total Bacillariophyceae; however, abundances were lower in the winters of 2013, 2016, and 2019 (January‒February). Chlorophyceae and Cyanophyceae, which had the second-highest abundances after Bacillariophyceae, were mainly abundant in summer. Although the Cryptophyceae abundance was only 0.9% of the total phytoplankton, the seasonal distribution of the dominant species, *Rhodomonas* sp., was an important factor in explaining the winter dominance of *C. vicinus*. However, their abundance was lower in autumn (i.e., November‒December) of 2015 and 2018 than in autumn of other years, similar to *Cyclotella* sp.

### 3.4. Growth Experiment

**Figure 8:**
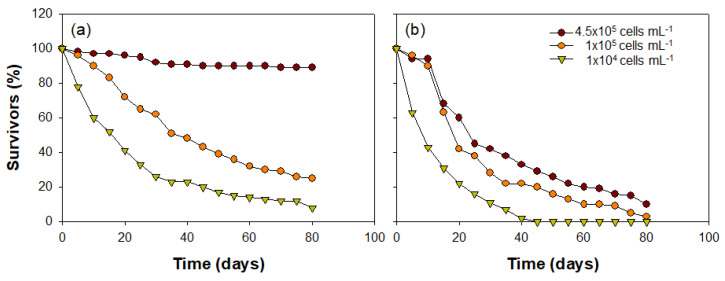
*Cyclops vicinus* survival (%) from nauplii to adult stage at three different concentrations of two food algae species: (**a**) *Cyclotella* sp. and (**b**) *Rhodomonas* sp.

The *C. vicinus* growth patterns with the various concentrations of food algae differed significantly depending on the algae species (*Cyclotella* sp. or *Rhodomonas* sp.) (Figure 8). Most nauplii developed into the adult phase under the high *Cyclotella* sp. concentration (4.5 × 10^5^ cells mL^−1^; approximately 97%), while the survival rate gradually decreased over time in treatments with lower food concentrations (1 × 10^5^ cells mL^−1^ and 1 × 10^4^ cells mL^−1^). The number of nauplii decreased to less than half within 45 days of the onset of the experiment under the intermediate *Cyclotella* sp. concentration (1 × 10^5^ cells mL^−1^), and at the lower concentration (1 × 10^4^ cells mL^−1^), a less than 50% survival rate was observed 20 days earlier than the intermediate concentration. In contrast, the survival rate of nauplii that had *Rhodomonas* sp. injected as a food source gradually decreased at all food concentrations. Although the *C. vicinus* survival rate was greater at the higher concentration of *Rhodomonas* sp., it was <20% under all food concentrations after 80 days. Interestingly, *C. vicinus* survival did not decrease for 10 days under high or intermediate *Rhodomonas* sp. concentrations; however, it began to decrease suddenly after 15 days. At low food concentrations, the survival rate decreased significantly from the beginning of the experiment, and all individuals died within 40 days. *C. vicinus* survival rates in the three concentrations of both food algae species were significantly different (Table 3).

## 4. Discussion

### 4.1. Seasonal Distribution Patterns

Cyclopoid copepods are frequently found in freshwater ecosystems (especially wetlands, ponds, and the middle-lower reaches of rivers and streams). They are primary consumers of phytoplankton or bacteria in freshwater food webs and are food sources for fish and invertebrates [55]. Thus, information on their distribution and ecological characteristics is important in terms of understanding the biological structure of freshwater ecosystems and securing ecosystem health. In this study, three cyclopoid copepod species (*C. vicinus*, *M. leuckarti*, and *Thermocyclops* sp.) were identified as dominant species. These species are widely distributed internationally, and their seasonal distribution and food preferences have been reported in various empirical studies [56,57,58]. These copepods are more abundant from spring to autumn, which is closely related to the ease of food acquisition and efficient population growth [15]. We found that *M. leuckarti* and *Thermocyclops* sp. were most abundant during summer‒autumn in the Upo Wetlands. Summer and autumn are beneficial for obtaining energy for spawning because water temperatures are high, and the water is also rich in food sources such as phytoplankton, which increase metabolic activity. Therefore, summer and autumn are the optimal seasons for copepod population growth and development since adequate supplies of food are available, and predators can be avoided.

Interestingly, we found that *C. vicinus* was more abundant during winter than during the general growth seasons (spring‒autumn) in the Upo Wetlands. The main reason why population growth in aquatic animals tends to be rarer in winter is that low water temperatures slow the metabolic rate and also make it difficult to acquire food, which greatly restricts growth and development [59]. In particular, zooplankton, including copepods, often have very low densities at low water temperatures [60,61]. However, we found that *C. vicinus* in the Upo Wetlands had a relatively long life cycle in winter. This can be attributed to a decrease in metabolic activity at low water temperatures. Nauplii and copepodites were mainly present from late autumn to early winter (late October‒ early December) and were supported by a high density of *C. vicinus* adults during most winter periods (December‒February). Given that nauplii and copepodites stages are bottlenecks in copepod development, the low nauplii and copepodite densities in most winter periods indicated that minimal *C. vicinus* reproduction occurs in winter. We speculate that in winter, *C. vicinus* adults come from the growth of nauplii and copepodites in autumn, which perform minimal reproductive activity during the winter and die off after laying resting eggs in February when water temperatures begin to increase. Based on the seasonal distribution pattern of *C. vicinus* domination every winter, the resting eggs appeared to remain dormant from spring to autumn and then hatched in late autumn. Although nauplii and copepodites were observed in spring (March‒May), these were considered to be the prior stages of *M. leuckarti* and *Thermocyclops* sp., which are mainly distributed in summer and autumn.

This winter distribution of *C. vicinus* would be an inefficient defense strategy if it were used solely to avoid relatively suitable environmental conditions (e.g., high metabolic activity and sufficient food) from spring to autumn. However, the spring‒autumn period in many locations has relatively high densities of other zooplankton communities, which consume similar food sources, as well as being the preferred growth season for predators such as fish which preferentially feed on *C. vicinus*. This makes spring‒autumn a difficult period for *C. vicinus* growth and indicates that winter may be an efficient period to avoid excessive interactions (competition or predation). In this location in South Korea, it is clear that the winter distribution of *C. vicinus* provides an evolutionary advantage to *C. vicinus* over other seasons. Changes in their seasonal distribution are closely related to the efficiency and persistence of population growth, which is the inherent nature of organisms. This is consistent with the evolutionary direction of almost all life on Earth.

### 4.2. Winter as a Temporal Refuge

In this study, *L. macrochirus*, a dominant fish species in the Upo Wetlands, consumed more copepods and cladocerans than any other food source. Previous studies have also suggested that *L. macrochirus* is a representative planktivorous fish, which mainly consumes zooplankton [62,63]. Cladocerans, which have relatively poor swimming capabilities, are preferred by fish predators over copepods, which leads to rapid depletion of cladoceran populations in the field [62]. Although copepods are also a frequent food source for fish, they can avoid predators more effectively because of their more rapid movements; therefore, consumption of copepods tends to occur after consumption of cladocerans [64]. This food shift in fish is an efficient predation strategy in response to seasonal distribution or density changes in the prey community. In this study, *L. macrochirus* consumed more cladocerans every spring than copepods; however, their choice changed to concentrated consumption of copepods in summer and autumn. Choi et al. [63] suggested that the dominance of *L. macrochirus* in wetlands in this area may lead to the near extinction of pelagic cladocerans. Pelagic cladocerans have a relatively large body size and frequent movements, making them relatively easy for fish such as *L. macrochirus* to locate and capture. We also found pelagic cladoceran species such as *Simocephalus vetulus* and *Daphnia* sp. in the gut contents of *L. macrochirus* during spring. Owing to the selective consumption of pelagic cladocerans, high densities of epiphytic cladocerans (*Chydorus*, *Alona,* and *Pleuroxus*) are found in the summer and autumn in most wetlands in South Korea. These species are difficult for *L. macrochirus* to consume.

The consumption of cyclopoid copepods by *L. macrochirus* in summer and autumn was mainly focused on *C. vicinus*. This selective consumption explains the low *C. vicinus* densities in summer and autumn. We assume that the higher consumption levels of *C. vicinus* by *L. macrochirus*, compared with other cyclopoid copepods (*M. leuckarti* and *Thermocyclops* sp.), is due to their ease of discovery. *Cyclops vicinus* has a larger body size relative to *M. leuckarti* and *Thermocyclops* sp., and hence, it can be prioritized during *L. macrochirus* foraging activities. The size of *Cyclops* spp. females ranges from 1.2 to 1.8 mm, while female *M. leuckarti* and *Thermocyclops* sp. range in size from 0.9 to 1.0 mm and from 0.7 to 0.8 mm, respectively [65]. Although empirical studies have suggested that dense aquatic macrophyte cover in summer and autumn can be used as a refuge for zooplankton to avoid fish predation, this is more effective for smaller species such as rotifers and cladocerans [66,67]. The space between the leaves and stems of aquatic macrophytes may also be an adequate refuge for smaller copepods, such as *M. leuckarti* and *Thermocyclops* sp. [68]; however, with relatively large bodies, *C. vicinus* is vulnerable to predators even in areas where aquatic macrophytes are abundant. This is likely to contribute to selective food consumption by *L. macrochirus* in the Upo Wetlands, where aquatic macrophytes are dominant.

Based on this information, it is logical to assume that the high consumption of *C. vicinus* by *L. macrochirus* may have induced the greater winter population growth of *C. vicinus*. Given that consumption of *C. vicinus* by *L. macrochirus* was observed to be constant from spring to autumn, it is evident that *C. vicinus* is also present in spring‒autumn in the Upo Wetlands. However, following the decline in cladoceran populations in summer and autumn, the concentrated consumption of copepods by *L. macrochirus* interrupts the stable population growth of *C. vicinus*. In this regard, winter is the only season that *C. vicinus* is able to avoid predation by fish. Fish, including *L. macrochirus*, have a low metabolic rate under low water temperatures, making it difficult to carry out efficient feeding activities during winter. Choi and Kim [29] and Speers-Roesch et al. [69] suggested that *L. macrochirus* is usually concentrated in withered vegetation during winter and that foraging activity and movement are minimal. Thus, the absence of predators in winter is advantageous for *C. vicinus*, which is strongly influenced by fish predation from spring to autumn. From this finding, we suggest that winter serves as a temporal refuge for stable population growth of *C. vicinus*.

### 4.3. Influence of Food Algae on Winter Distribution of C. vicinus

In the Upo wetlands, phytoplankton were mainly abundant in winter because of the seasonal variability of Bacillariophyceae, i.e., a dominant species of phytoplankton. Most wetlands located in Korea, including the Upo Wetlands, are almost completely covered by aquatic macrophytes from spring to autumn, which reduces light entering the water and strongly restricts phytoplankton growth because of nutrient competition with aquatic macrophytes [70]. Winter supports a lower aquatic macrophyte abundance and bountiful nutrients, which makes it a suitable season for Bacillariophyceae growth even in low water temperatures [71]. Thus, winter dominance of Bacillariophyceae is found in various regions, including Korea [71,72,73].

We focused on the fact that the Bacillariophyceae distribution was supported by different species composition in the winter of every year. Interestingly, the difference in the species composition of Bacillariophyceae was closely related to the *C. vicinus* distribution in winter. In years when the winter peak of *C. vicinus* was observed (January‒February in 2014, 2015, 2017, and 2018), *Cyclotella* sp. accounted for >80% of total Bacillariophyceae, while the *C. vicinus* density was lower in years in which there was a lower *Cyclotella* sp. abundance (January‒February in 2013, 2016, and 2019). Winter water temperature is an important factor in determining the winter distribution of *Cyclotella* sp., and we found that the *Cyclotella* sp. abundance and *C. vicinus* density were low in years when water temperatures remained relatively high in winter (>5 °C; 2013, 2016, and 2019). The matching temporal distributions of *Cyclotella* sp. and *C. vicinus* indicate that *Cyclotella* sp. is closely related to the winter distribution of *C. vicinus*. Santer [74] suggested that cyclopoid copepods such as *Eudiaplomus gracilis*, and *M. leucarti* prefer food algae with soft shells, while *Cyclops abyssorum* may consume the dinoflagellate *Ceratium furcoides,* which has a hard shell. *C.* *vicinus*, which is able to feed on prey with hard shells, can grow in winter, unlike *M. leuckarti* and *Thermocyclops* sp., because *Cyclotella* sp. is available as a food source. The results of stable isotope analysis provide a physical basis for the use of *Cyclotella* sp. as a winter food source for *C. vicinus*. The SPOM, supported primarily by phytoplankton, clearly contributed as a food source for *C. vicinus* in winter (January‒February in 2014, 2015, 2017, and 2018), when *Cyclotella* sp. was abundant, but had a weaker association with SPOM in the winters of 2013, 2016, and 2019, when *Cyclotella* sp. was not dominant in the phytoplankton community. Moreover, the drastic decrease in *C. vicinus* in February 2018 can also be attributed to the low *Cyclotella* sp. abundance. The rapid increase in water temperature during this period caused the depletion of *Cyclotella* sp.; this association was also found in *Rhodomonas* spp. belonging to Cryptophyceae. Although the *Rhodomonas* sp. abundance was low during winter, high density was observed in late autumn (October–November), just before winter began. We assumed that the late autumn growth of *Rhodomonas* sp. influenced the germination and growth of nauplii and copepodites. We found that *Rhodomonas* sp. was abundant in the late autumn of the previous year when the winter dominance of *C. vicinus* was observed in January‒February in 2014, 2015, 2017, and 2018.

Although we surmised that *Rhodomonas* sp. did not play an important role in the winter distribution of *C. vicinus*, it contributed as a food source in the early growth stages of *C. vicinus* (i.e., nauplii stage). Growth experiments using microcosms showed that the *Rhodomonas* sp. concentration did not significantly affect *C. vicinus* growth. Nauplii survived for nearly 10 days at concentrations of 4.5 × 10^5^ cells mL^−1^ and 1 × 10^4^ cells mL^−1^ of *Rhodomonas* sp. (i.e., excluding low concentrations); however, their survival rate declined sharply from 15 days onward. Under the low food concentration, survival rates dropped sharply from the beginning of the experiment, and all individuals were dead within 40 days. This means that *Rhodomonas* sp. is rarely used as a suitable food source in the growth stage of *C. vicinus* after 10 days. This result was clearly different in the case of *Cyclotella* sp., in which survival rates rarely decreased under high food concentrations. From these results, we concluded that the concentration and type of food algae strongly influence *C. vicinus* growth. Hopp and Maier [75] also suggested that *C. vicinus* nauplii had a very high mortality rate under low food algae concentration conditions, whereas *M. leuckarti* and *Thermocyclops crassus* were not significantly affected by the food concentration. The summer diapause of *Cyclops* spp., which is frequently observed in Europe, is interpreted as a strategy caused by competition or food shortages among the nauplii stages [76]. In general, cyclopoid copepods vary in their preferred food conditions depending on the species and developmental stage; therefore, seasonal or annual changes in food sources and food availability can strongly affect the seasonal distribution of copepods [77,78]. For example, Santer and Van an den Bosch [79] explained that food algae, such as phytoflagellates, are effective in the nauplii or copepodite stages but are rarely utilized in the adult stage.

Although the results are not presented here, we suggest an additional possibility based on previous studies. The rotifers distributed in late autumn may have affected the winter distribution of *C. vicinus*. Cyclopoid copepods, which are distributed in spring in various regions, frequently consume rotifers as a food source. Devetter and Seďa [80] suggested that spring copepods preferred soft-bodied rotifer species (e.g., *Synchaeta* sp. and *Polyartra* sp.), while hard-bodied rotifer species (*K. cochlearis* and *K. longispina*) were consumed more slowly. Choi et al. [81] found that the rotifer density was low in winter but high in late autumn (October‒November). Although *C. vicinus* is mainly distributed at the nauplii or copepodites stages in late autumn, the possibility of consumption cannot be ruled out. The effects of rotifers as a food source on *C. vicinus* require further investigation.

### 4.4. Evolution of Refuge Utilization of Prey to Maintain Sustainable Populations

We surmised that the winter *C. vicinus* populations diverged from the community distribution from spring to autumn. High foraging activities during spring‒autumn, owing to the establishment of exotic fish species such as *L. macrochirus*, required a new survival strategy for stable *C. vicinus* population growth. We assumed that the winter distribution of copepods, such as the hypolimnion and vegetated areas, is an evolved form of refuge utilization. The zooplankton refuges reported in previous studies include moving to a specific space or concentrating in one region, based on predators’ habitat preferences. Thus, the presence of predator-avoidance spaces is important for prey such as zooplankton. However, these previous refuges do not have adequate conditions as habitats for zooplankton. It is difficult for phytoplankton, which are food sources for zooplankton, to grow in the hypolimnion and vegetated areas because of the low light, and these places offer inconsistent refuge efficiency owing to seasonal or regional differences in growth. Although these spaces are somewhat suitable for avoiding predators, they have long-term environmental conditions that are adverse for the stable growth of prey populations. From this point of view, winter is an efficient temporal refuge for the copepod *C. vicinus*, providing adequate food sources and effective predator avoidance. The use of this temporal refuge by *C. vicinus* is an evolutionary defense strategy to resist the predatory activities of *L. macrochirus*, which has developed to enable efficient food acquisition in areas with a high aquatic macrophyte cover. *L. macrochirus* has also evolved to use aquatic macrophytes as refuge to avoid predation by *M. salmoides* [29,63]. Currently, *C. vicinus* is positioned at a higher trophic level than its main predator through an evolved defense strategy—winter distribution. In terms of the evolutionary arms race, a predator’s foraging efficiency will need to adapt to the evolution of its prey. However, provided other prey species are present from spring to autumn, *L. macrochirus* survival will not be threatened, and there may not be a reason to choose the poor conditions of winter distribution. It is clear that *C. vicinus* will increase its chances of survival by actively utilizing this temporal refuge and also produce more offspring through stable population growth. This evolution of prey defense strategies against predators increases local biodiversity and affects the stable interaction between aquatic organisms in freshwater food webs. Based on the discovery of this new form of refuge, it is necessary to investigate the presence of evolutionary strategies of predators or/and prey in areas where prey–predator interactions are frequent.

## 5. Conclusions

A habitat-related disadvantage of *C. vicinus*, which are significantly larger species than other cyclopoid copepods, results in higher predation pressure due to a higher chance of being detected by the main fish predators in the shared summer habitat, which has dense vegetation and low visibility. The fish predator, *L. macrochirus*, is well adapted to the summer habitat and detects, encounters, and ingests prey most frequently. However, fish predators are seriously impaired in winter temperatures and thus are unable to compete in the predator‒prey arms race. Furthermore, winter offers a stable uncontested food opportunity—phytoplankton abundance that is different from the warm season and specific to the cold season. Culture experiments showed that *Cyclotella* sp. contributed more to the growth stage (copepodite or subadult) after nauplii than *Rhodomonas* sp. From this finding, we assume that the timing of peak reproduction and abundance of *C. vicinus* has changed from the warm to the cold season.

## Figures and Tables

**Table 1:** Seasonal changes in diet composition (ind. gut weight^−1^) of *Lepomis macrochirus* in the study site in the Upo Wetlands.

Year	Season	Copepods	Branchiopods	Isopods	Dipterans	Odonatans	Young Fish
2014	Winter	-	-	-	-	-	-
	Spring	10.2 ± 3.7	22.5 ± 2.8	6.2 ± 4.1	8.3 ± 2.5	3.4 ± 1.2	-
	Summer	22.1 ± 5.1	10.7 ± 6.7	5.8 ± 1.9	9.1 ± 4.2	5.6 ± 2.3	-
	Autumn	18.7 ± 6.2	13.2 ± 4.8	5.3 ± 1.2	9.2 ± 3.4	5.4 ± 3.7	0.6 ± 0.1
2015	Winter	-	-	-	-	-	-
	Spring	12.1 ± 2.9	26.1 ± 8.4	3.8 ± 1.1	10.2 ± 3.4	6.2 ± 2.1	-
	Summer	26.4 ± 8.2	13.1 ± 9.0	7.2 ± 2.5	11.8 ± 6.4	5.2 ± 2.7	-
	Autumn	23.4 ± 7.4	10.7 ± 8.1	6.0 ± 3.5	7.6 ± 2.8	5.7 ± 3.7	-
2016	Winter	4.5 ± 1.1	2.3 ± 1.2	-	-	-	-
	Spring	15.2 ± 4.8	23.7 ± 6.4	4.2 ± 1.4	8.6 ± 2.8	5.3 ± 1.4	-
	Summer	25.4 ± 9.2	11.5 ± 3.8	6.2 ± 2.1	10.4 ± 2.8	6.8 ± 2.1	-
	Autumn	27.8 ± 10.3	13.2 ± 7.2	5.8 ± 3.5	12.7 ± 5.2	6.4 ± 1.8	-
2017	Winter	-	-	-	-	-	-
	Spring	16.3 ± 7.4	26.1 ± 8.2	6.2 ± 2.4	8.1 ± 2.3	4.2 ± 1.1	
	Summer	24.1 ± 7.3	10.6 ± 3.2	8.4 ± 3.1	10.2 ± 5.2	6.2 ± 4.2	0.5 ± 0.2
	Autumn	28.1 ± 11.3	15.2 ± 8.2	7.4 ± 5.2	10.8 ± 4.7	5.6 ± 1.8	0.7 ± 0.3
2018	Winter	-	1.1 ± 0.7	-	-	-	-
	Spring	10.3 ± 5.2	23.1 ± 10.2	5.3 ± 2.0	12.4 ± 4.6	7.2 ± 2.1	-
	Summer	27.2 ± 9.4	13.4 ± 6.8	10.4 ± 2.5	10.3 ± 3.9	6.3 ± 1.4	-
	Autumn	20.4 ± 8.2	13.1 ± 6.2	7.3 ± 3.6	7.8 ± 1.9	8.3 ± 2.6	-
2019	Winter	6.1 ± 2.1	1.6 ± 0.2	-	-	-	-

**Table 2:** Seasonal changes of dominant cyclopoid copepods in the diet (ind. gut weight^−1^) of *Lepomis macrochirus* in the study site in the Upo Wetlands.

Year	Season	*Cyclops vicinus*	*Mesocyclops leuckarti*	*Thermocyclops* sp.
2014	Winter	-	-	-
	Spring	6.2 ± 2.1	2.3 ± 0.5	1.7 ± 0.2
	Summer	11.2 ± 3.5	7.2 ± 1.8	3.7 ± 1.1
	Autumn	8.6 ± 2.7	6.4 ± 1.3	3.7 ± 0.9
2015	Winter	1.1 ± 0.4	-	-
	Spring	6.2 ± 1.7	3.2 ± 1.3	2.7 ± 0.7
	Summer	12.4 ± 3.7	8.7 ± 2.3	5.3 ± 1.0
	Autumn	12.4 ± 2.8	7.6 ± 1.7	3.4 ± 0.8
2016	Winter	4.5 ± 1.1	-	-
	Spring	6.4 ± 2.4	5.2 ± 1.4	3.6 ± 1.3
	Summer	11.3 ± 2.8	9.9 ± 2.9	4.2 ± 0.7
	Autumn	12.4 ± 3.6	8.4 ± 2.7	7.0 ± 2.8
2017	Winter	-	-	-
	Spring	8.4 ± 2.5	4.3 ± 1.5	3.6 ± 1.3
	Summer	11.4 ± 4.1	8.4 ± 2.7	4.3 ± 0.8
	Autumn	13.6 ± 2.6	8.6 ± 3.4	7.9 ± 1.3
2018	Winter	-	-	-
	Spring	5.2 ± 1.6	3.7 ± 0.4	1.4 ± 0.3
	Summer	12.4 ± 4.3	10.7 ± 2.9	4.1 ± 0.7
	Autumn	9.4 ± 1.8	6.8 ± 2.7	4.2 ± 1.4
2019	Winter	6.1 ± 2.1	-	-

**Table 3:** Two-way nested analysis of variance (ANOVA) results for the effects of main groups (i.e., three food concentrations; 1 × 10^4^ cells mL^−1^, 1 × 10^5^ cells mL^−1^, and 4.5 × 10^5^ cells mL^−1^) and subgroups (i.e., 20 beakers) on *Cyclops vicinus* survival (%).

Food Type	Variance	d.f.	F	*p*
*Cyclotella* sp.	Food concentrations	2	92.478	*p* < 0.001
	Beaker	18	0.168	*p* > 0.05
*Rhodomonas* sp.	Food concentrations	2	8.214	*p* < 0.05
	Beaker	18	0.208	*p* > 0.05

## Data Availability

The data presented in this study are available on request from the corresponding author. The data are not publicly available due to restrictions on the right to privacy.
